# Deletion of Thioredoxin Reductase and Effects of Selenite and Selenate Toxicity in *Caenorhabditis elegans*

**DOI:** 10.1371/journal.pone.0071525

**Published:** 2013-08-06

**Authors:** Christopher J. Boehler, Anna M. Raines, Roger A. Sunde

**Affiliations:** Department of Nutritional Sciences, University of Wisconsin-Madison, Madison, Wisconsin, United States of America; Instituto de Biociencias - Universidade de São Paulo, Brazil

## Abstract

Thioredoxin reductase-1 (TRXR-1) is the sole selenoprotein in *C. elegans*, and selenite is a substrate for thioredoxin reductase, so TRXR-1 may play a role in metabolism of selenium (Se) to toxic forms. To study the role of TRXR in Se toxicity, we cultured *C. elegans* with deletions of *trxr-1*, *trxr-2*, and both in axenic media with increasing concentrations of inorganic Se. Wild-type *C. elegans* cultured for 12 days in Se-deficient axenic media grow and reproduce equivalent to Se-supplemented media. Supplementation with 0–2 mM Se as selenite results in inverse, sigmoidal response curves with an LC_50_ of 0.20 mM Se, due to impaired growth rather than reproduction. Deletion of *trxr-1*, *trxr-2* or both does not modulate growth or Se toxicity in *C. elegans* grown axenically, and ^75^Se labeling showed that TRXR-1 arises from the *trxr-1* gene and not from bacterial genes. Se response curves for selenide (LC_50_ 0.23 mM Se) were identical to selenite, but selenate was 1/4^th^ as toxic (LC_50_ 0.95 mM Se) as selenite and not modulated by TRXR deletion. These nutritional and genetic studies in axenic media show that Se and TRXR are not essential for *C. elegans*, and that TRXR alone is not essential for metabolism of inorganic Se to toxic species.

## Introduction

Selenium (Se) is an essential trace element in mammals but Se is also toxic to bacteria, yeast, plants and animals, including *C. elegans*. Concentrations >2 mM Se block growth in bacteria and yeast [1], [2], and are lethal in short-term acute studies with adult *C. elegans* [3]. In animals, selenite and selenate toxicity is observed at >20X the dietary Se requirement [4], [5]. In humans, adverse effects of Se occur at 20X the recommended dietary allowance (RDA) after long-term exposure [6], and acute toxicity has even occurred in the US recently due to misformulated health supplements that provided up to 740X the RDA [7]. Rodent cancer studies and human epidemiological and clinical trials, however, have shown that Se supplementation at levels 4–5X the RDA protects against a variety of cancers [8]–[10], whereas selenium supplementation is also associated with increased risk of cardiovascular disease and diabetes [11]–[13]. Clearly a better understanding of Se toxicity is needed.

Se is essential because it is incorporated as selenocysteine (Sec) during translation into the peptide backbone of a small set of selenoproteins, at the position specified by a UGA codon and mediated by a unique tRNA [14]. Common inorganic forms of Se, selenite and selenate, are readily reduced to selenide which is used for this cotranslational Sec synthesis and incorporation into selenoproteins [15], [16]. Se toxicity is thought to be associated with reduced forms of Se. The initial metabolism of selenate to selenite occurs via the sulfate reduction pathway, and selenite is a substrate for thioredoxin reductase which directly releases selenide as the product [17]. Thus thioredoxin reductase may play an important role in Se metabolism including formation of toxic forms of Se.

Humans have a total of 25 selenoproteins. All three mammalian thioredoxin reductases, TXNRD1, TXNRD2, TXNRD3, are selenoproteins [18], and deletion of TXNRD1 or TXNRD2 in mice results in embryonic lethality [19]. The *C. elegans* genome encodes two thioredoxin reductases. Thioredoxin reductase-1 (TRXR-1) is the sole selenoprotein in *C. elegans*, with a UGA-encoded Sec in the C-terminal active site [20], [21]. The second thioredoxin reductase, TRXR-2, is a homolog with a UGU encoded cysteine substitution for Sec. In contrast to at least mice, Stenvall et al [22] showed recently that deletion of *trxr-1*, *trxr-2*, or both in *C. elegans* was without phenotype under standard conditions, raising the question of why *C. elegans* has retained genes for TRXR-1, the Sec-tRNA and the other components of the Se insertion mechanism. A role for TRXR-1 in Se metabolism might explain this conservation.

To study the role of TRXR in protection against Se-induced toxicity, we cultured *C. elegans* strains with deletions of *trxr-1*, *trxr-2*, or both (*dko*) with increasing concentrations of different inorganic forms of Se. We used axenic media, eliminating bacterial Se metabolism, to show Se is not a required nutrient for *C. elegans* under standard conditions. We found that *trxr-1*(*−/−*), *trxr-2*(*−/−*) and *dko* strains grow and develop normally in axenic media, that selenate is 1/4 as toxic as selenite or selenide, and that deletion of *trxr-1*, *trxr-2*, or both does not alter susceptibility to Se toxicity for any of these forms of inorganic Se.

## Materials and Methods

### Reagents

Molecular biology reagents were purchased from Promega (Madison, WI), Invitrogen (Carlsbad, CA) or Sigma (St. Louis, MO). All other chemicals were of molecular biology or reagent grade.

### Strains, Maintenance, Growth Conditions

**Figure 1:**
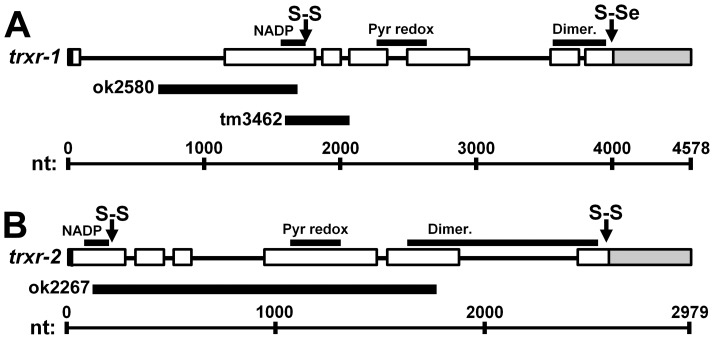
Thioredoxin reductase deletions. For *trxr-1* (A) and *trxr-2* (B), open boxes indicate exons, shaded gray boxes indicate untranslated regions (UTR) and lines indicate introns for each strain. Catalytic disulfides (S-S), selenosulfides (S-Se), Rossmann-fold NADP binding domains (NADP), pyridine nucleotide disulfide domains (Pyr redox) and pyridine nucleotide disulfide dimerisation domains (Dimer.) are indicated in the above diagrams. Black bars below the gene diagrams show deletions in deletion strains.

The wild-type *C. elegans* strain used in all experiments was N2 Bristol (N2) obtained from the Caenorhabditis Genetic Center (CGC, Minneapolis, MN). *Trxr-1* deletion mutant RB1961 *trxr-1* (ok2580) and *trxr-2* deletion mutant RB1764 *trxr-2* (ok2267) were produced by the *C. elegans* Gene Knockout Consortium Project and obtained from the CGC. *Trxr-1* deletion mutant FX03462 *trxr-1* (tm3462) was obtained from the National Bioresource Project for the Nematode (Dr. Shohei Mitani, Tokyo Women's Medical University School of Medicine, Shinjuku-ku, Tokyo, Japan). Strains were outcrossed at least 6X with N2 males. Additionally, a double knock-out strain was generated by crossing ok2580 and ok2267. The location of each deletion was mapped by sequencing of PCR products spanning the deleted region (**Fig. 1**). ok2580 has a 1023 bp deletion of *trxr-1* on chromosome IV from 7012654–7013677 removing portions of exon 2 and the NADP-binding domain. tm3462 has a 474 bp deletion from 7013585–7014059, removing exons 2 and 3 containing the catalytic disulfide and the NADP-binding domain of *trxr-1*. ok2267 has a 1645 bp deletion of *trxr-2* on chromosome III from 8913764–8915409 spanning exons 1–5, removing the catalytic disulfide, NADP-binding and pyridine-disulfide oxidoreductase domains.

Maintenance and growth of *C. elegans* is started initially under standard conditions on nematode growth media (NGM) agar plates with an *E. coli* OP50 lawn [23]. Following growth to gravid (egg-bearing) adults, *C. elegans* are bleached in 1.1% Clorox bleach (sodium hypochlorite)/0.55 M NaOH to release eggs, as described by Rao et al. [24]. Eggs are hatched overnight in M9 buffer to produce a synchronized population of growth-arrested L1 larvae. L1 *C. elegans* are then inoculated into cultures of CeHR-3 media, a defined liquid axenic developed by Iqbal Hamza's group at the University of Maryland [24]. Our basal axenic media contains 0.000125 mM Se, kindly determined by Steve Morris at the University of Missouri Research Reactor (Columbia, MO).

### Selenium Dose Response Curves

**Figure 2:**
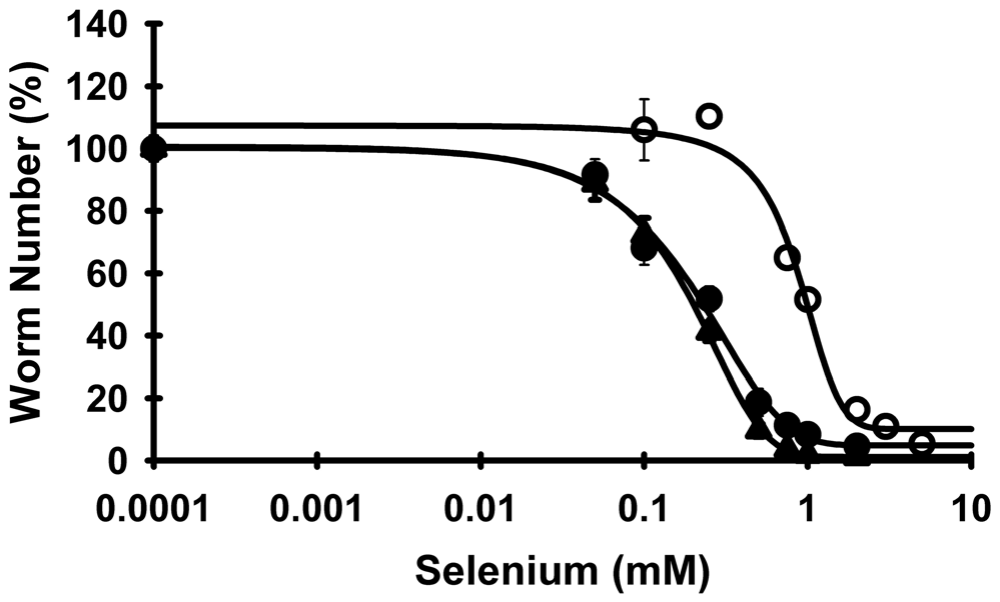
Selenium response curves for selenite, selenide and selenate. Se toxicity for selenite, selenide, and selenate was assessed in N2 *C. elegans* grown in axenic media for 12 days and supplemented with 0–2 mM Se as selenite (▴) and selenide (•), and with 0–5 mM Se as selenate (○). Initial seeding was 100 L1/well, and total worm number in the mixed cultures was counted at day 12. Values are the means ± SEM for 3 replicates at each concentration, and solid lines are 4-parameter sigmoidal regression lines for each form of Se. The LC_50_ (Se concentration that results in 50% of plateau number of worms) for selenate was 0.95 mM Se and significantly different from selenite and selenide (0.20 and 0.23 mM Se, respectively), as shown in Table 1.

L1 larvae were inoculated into 10 mL (100 L1/mL) of axenic media and grown for 6–7 days with shaking (70 rpm) at 20°C. Resulting gravid *C.elegans* were then treated with bleach/NaOH solution, eggs hatched overnight, and resulting L1s were used to inoculate 1 mL/well cultures (100 L1/well) of axenic media in 24-well plates (Fisher, Waltham, MA), containing graded concentrations of Se from 0–5 mM Se as either sodium selenite, sodium selenate, or sodium selenide (Sigma) (3 wells/concentration). Cultures were allowed to grow and reproduce for 12 days. Total numbers of worms at day 12 were determined by anesthetizing worms with 10 mM sodium azide and counting all worms in 3×20 µL aliquots of the mixed cultures (L1 to adult) for each well using a Zeiss Stemi 2000 dissecting scope (**Fig. 2**).

### Daily growth and development

Synchronized, N2 L1's were inoculated at 100 L1/well in CeHR-3 media supplemented with 0, 0.05, or 0.2 mM Se as sodium selenite. Worms were removed from an individual well daily to quantitate length by anesthetizing worms on a 3% agarose pad on a microscope slide and capturing images with a Zeiss Axioplan 2 microscope equipped with a Zeiss AxioCam MRm digital camera. Length of individual worms was measured using Image J software (NIH) by drawing a medial line from nose to tail tip for worms at the predominant developmental life cycle [25] stage.

### Brood size determination

Synchronous N2 10-mL cultures of L1s were counted and diluted to provide 5 L1/well (1 mL) of axenic media with 0, 0.05, or 0.2 mM Se as sodium selenite, in triplicate. Actual starting worms/well ranged from 2–7. Non-adult worms (offspring) were counted daily until a plateau was reached but before offspring reached adulthood (up to 15 days for 0.2 mM Se). This plateau offspring number/well was divided by starting worm number to determine brood size.

### ^75^Se-labeling of TRXR-1

Synchronous L1 larvae (100/mL) were inoculated into 10 mL axenic media and grown for 6 d. *C. elegans* were pelleted (800× g×5 min at 20°C, Beckman X-12R, SX4750 rotor, Palo Alto CA), added to 50 mL axenic media containing 1 µCi/mL [^75^Se]selenite (489 µCi/ug Se, University of Missouri Research Reactor, Columbia MO) and grown for 12 days. Resulting *C. elegans* were pelleted (5°C) and resuspended three-times in PBS (50 mM sodium PO_4_, 75 mM NaCl, 1 mM EDTA, pH 7.4) to remove unbound ^75^Se. *C. elegans* were again pelleted, supernatant rapidly removed, and the pellet frozen at −80°C for later analysis. Frozen cultures were thawed, disruption buffer added to make 20% homogenates (final homogenate contained 25 mM Tris, 1 mM EDTA, 1 mM PMSF, 1 mM DTT, pH 7.6), sonicated at 8 W for 10 sec (60 Sonic Dismembrator, Fisher), and immediately placed on ice before centrifugation (13,800× g×30 min, 5°C, Eppendorf 5415R). The resulting supernatants were analyzed for protein [26], and subjected to SDS-PAGE analysis on 10% acrylamide gels as described previously [27]. After electrophoresis, gels were stained, destained, dried, and selenoprotein labeling analyzed by autoradiography (Kodak Biomax XAR-5, Rochester, NY) as described previously [27].

### TRXR enzyme activity

Fifty mL cultures of *C. elegans* were grown in media containing 0, 0.05, or 0.1 mM Se as selenite, harvested, and frozen as described for ^75^Se labeling studies. At least 3 separately grown and harvested replicates for each strain were thawed and assayed at the same time. Supernatants resulting from the sonication of 20% homogenates were dialyzed against PBS overnight (1 change of buffer) to remove low molecular weight thiols, centrifuged (13,800× g×30 min, 5°C), heat denatured at 50°C for 10 min, and centrifuged again to prepare heat-denatured supernatants for TRXR enzyme activity measurements. After analysis for protein, each supernatant (50 µg protein) was assayed in triplicate with and without human thioredoxin (TXN) (T8690, Sigma) for 10 min using a modified procedure [28] of that described by Holmgren and Bjornstedt [29]. Briefly, assay tubes containing 195 µl of reaction mix were pre-incubated at 37°C for 5 min before 55 µl of 37°C supernatant (50 µg protein) were added, resulting in a final concentration of 10 mM HEPES pH 7.6, 5 mM EDTA, 0.8 mM NADPH, 2.14 µg insulin/ul, and 2.5 uM human TXN. Each reaction was accompanied by a duplicate tube lacking TXN, so txn-dependent activity was determined. Assays were stopped after 10 min by addition of 750 µl of freshly prepared stop mix (5.4 N guanidinium, 1.8 M Tris, pH 8.0, containing 1 mM DTNB), and reduced DTNB absorbance was measured at 412 nm. One enzyme unit is defined as the TXN-specific reduction of 1 µmole of DTNB in 10 min under these conditions. Activity was also assessed in *trxr-2*(*−/−*) and *dko* strains grown with 0.05 mM Se.

### Glutathione reductase (Gsr) enzyme activity

Heat-denatured supernatants were also assayed for GSR activity as described by Massey and Williams [30]. One enzyme unit is defined as the quantity that will oxidize 1 µmole of NADPH per minute under these conditions.

### qRT-PCR

*Trxr-1*, *trxr-2* and *gsr-1* transcript levels were assessed by qRT-PCR as described previously [31]. Briefly, total RNA was isolated with TRIzol reagent (Invitrogen, Carlsbad, CA) from dense, mixed staged N2 cultures grown in axenic media supplemented with 0, 0.025, 0.05, 0.1, and 0.25 mM Se as selenite. One µg of total RNA was reverse transcribed to cDNA using the RETROscript kit (AM1710, Ambion, Austin, TX, U.S.A.), following the manufacturer's instructions. Gene specific primers for *trxr-1* were: sense primer 5′-CCGAGTTATTCGGTTGCTTC-3′, antisense primer 5′-ATTCCACTCGCTGCTACTCC-3′; *trxr-2* sense primer 5′-TCGTCGGGAAAGAGCTAAAA-3′, antisense primer 5′-ATCCACAAACTCGGCATAGG-3′; *gsr-1* sense primer 5′-CGGATTTGATGTGACGCTTA-3′, antisense primer 5′-AAAGTTGCACGTCCTCGAAT-3′. The final real time reactions containing reverse transcribed RNA, gene-specific forward and reverse primers and 1X SybrGreen PCR Master Mix (#4309155, Applied Biosystems, Foster City, CA, U.S.A.) were performed in an ABI Prism 7000 Sequence Detection System (Applied Biosystems), with initial stages of 50°C for 2 min and 95°C for 10 min, followed by 50 cycles of 95°C for 15 sec and 60°C for 2 min. A dissociation curve was run for each plate to confirm production of a single product. The amplification efficiency for each gene was determined using the DART program. Relative abundance of each mRNA was calculated using the method of Pfaffl [32], accounting for gene specific efficiencies, normalized to eft-3, and set to the relative mean of the 0.025 mM Se concentration.

### Statistical Analysis

Statistical analysis was performed using R version 2.13.0 software. Results are presented as the mean ± SEM. Selenium dose response curves were generated using a 4-parameter sigmoidal regression (Sigmaplot 6.0, Jandel Scientific, San Rafael, CA), and each curve represents at least 3 independent experiments with 9 replicates at each concentration. The 50% lethal concentration (LC_50_) was determined graphically as the Se concentrations on the Se response curve resulting in 50% of plateau level worm number. Differences in LC_50_ values, TRXR, GSR activity levels, daily growth, and brood sizes across strains and Se concentrations were analyzed by a one-way analysis of variance (ANOVA), followed by a protected LSD analysis for individual differences between strains. A repeated measures ANOVA was used to determine treatment, day, and treatment-day interaction differences in daily worm growth. Means with different letters are statistically different, *P*<0.05.

## Results

### Selenium is not essential but is toxic

Wild-type N2 *C. elegans* grown in Se-deficient, axenic media supplemented with graded-levels of selenium as selenite display an inverse, sigmoidal response (**Fig. 2**). In Se-deficient media, containing 0.0001 mM Se, there is no impairment in worm number as compared to *C. elegans* grown in 0.05 mM Se as selenite, indicating selenium is not required for proper growth and reproduction. At 1 mM Se, worms fail to develop to adults and by 2 mM Se no notable development beyond L1 is observed. In Fig. 2, the LC_50_ is 0.20 mM Se as selenite. Worms grown for several weeks in media containing 1 mM Se develop a red color which presumably is elemental Se [33]; these individuals do not develop to the L4 stage, and worms cultured in 2 mM Se develop the red color but do not develop past the L1 stage (data not shown).

### Decreased worm number with high Se results from slowed growth and not reproductive impairment

**Figure 3:**
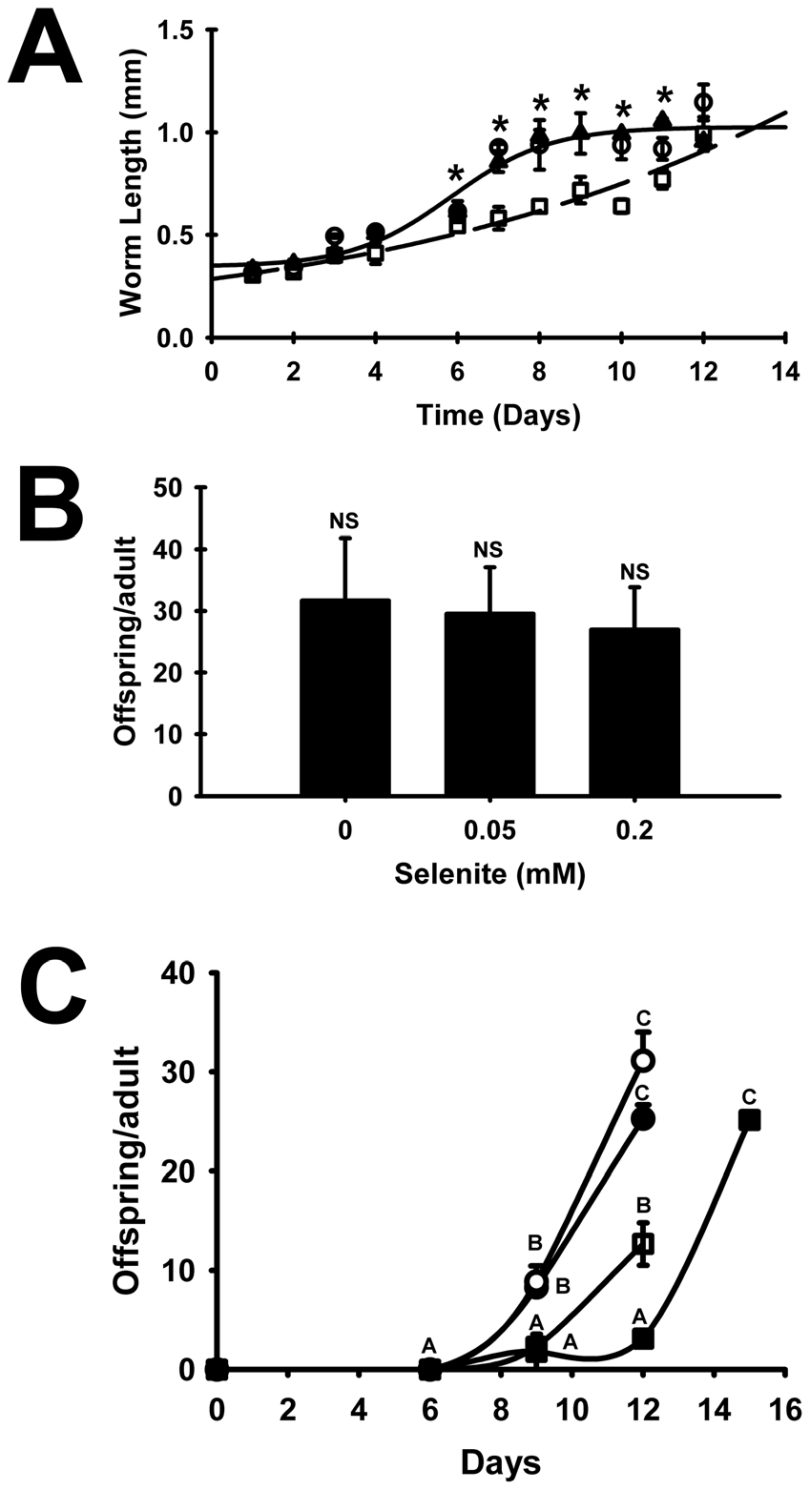
Effect of Se on *C. elegans* length and brood size. (A) Synchronized N2 L1s were inoculated (100/well) into axenic media (0.0001 mM) supplemented with 0 (▴), 0.05(○), or 0.2 (□) mM Se as Na_2_SeO_3_. Worm length was measured daily. Solid line shows the regression of 0 and 0.05 mM Se treatments combined, and dashed line shows regression of the 0.2 mM Se treatments. Values represents the mean ± SEM. Two-way ANOVA indicates significant effect of day (*P*<0.001), Se (*P*<0.001), and interaction (*P*<0.001); * indicates a significant difference from 0.2 mM Se treatment, P<0.05. (B) Synchronized N2 L1s were inoculated (2–7/well) into axenic media (0.0001 mM) supplemented with 0, 0.05, or 0.2 mM Se as sodium selenite. Average brood size/adult was assessed in triplicate wells for up to 15 days. Values represents the mean ± SEM (*P* = 0.686), NS indicates no significant difference. (C) Synchronized N2 L1s were inoculated at two densities, 5 L1/well (•,▪) or 100 L1/well (○,□), into axenic media supplemented with 0 mM Se (•,○) or 0.2 mM Se (▪,□) as selenite. Average offspring/adult was assessed in triplicate at days 6, 9, and 12, and also at day 15 for 5 L1/well worms grown in 0.2 mM Se. Values represents the means ± SEM. A, B, C: Means with different letters are statistically different, *P*<0.05.

To determine the effect of increasing media Se on worm growth, N2 L1s were grown for 12 days in axenic media supplemented with 0, 0.05, or 0.2 mM Se as selenite. Over the first 4 days, there was little difference in length with or without Se supplementation. Worms supplemented with 0 or 0.05 mM Se displayed nearly identical daily growth and worm number over the full 12 days (**Fig. 3A**). By day 7, worms supplemented with 0 or 0.05 mM Se were 45% longer than worms supplemented with 0.2 mM Se; by day 8, worms supplemented with 0 and 0.05 mM Se reached a plateau length of 1 mm whereas 0.2 mM Se supplemented worms were significantly shorter until day 12, indicating that Se toxicity causes impaired growth in *C. elegans*.

To further study the nature of the inverse relationship between worm number and increasing Se concentration, brood size from individual worms was assessed in Se-deficient axenic media supplemented with 0, 0.05, or 0.2 mM Se as selenite. Because of delayed growth with 0.2 mM Se, brood size for worms grown in 0.2 mM was determined at 15 days so full brood size was assessed. N2 grown in 0, 0.05 or 0.2 mM Se showed no significant differences in final progeny number (31.7±10.1, 29.5±7.5, 27.0±6.9, respectively; *P* = 0.69) (**Fig. 3B**).

To better understand the nature of worm number counted at day 12, we assessed offspring number at day 6, 9, and 12 in cultures seeded initially with 100 or 5 L1/well (**Fig. 3C**). In axenic media, there were no offspring at day 6. By day 9 for worms cultured without supplemental Se, there was an average of 10 offspring per initial L1 in wells seeded with 100 or 5 worms, suggesting that about 1/3^rd^ of original L1s had laid eggs. By day 12, offspring per initial L1 in 0 mM Se averaged about 30 regardless of initial seeding density and similar to the data in **Fig. 3B**. With 0.2 mM Se supplementation at day 9, there was an average of 2 offspring per initial L1 in wells seeded with 100 or 5 worms, indicating that less than 10% of original L1s had laid eggs. By day 12, with 100 L1/well seeding, offspring per initial L1 with 0.2 mM Se were about 50% of that observed without Se supplementation similar to the effect of selenite supplementation shown in **Fig. 2**. At day 12, with 5 L1/well seeding, offspring per initial L1 with 0.2 mM Se were little different from day 9, but rose to near 30 offspring per initial L1 by day 15, similar to the experiment in **Fig. 3B**. Collectively these studies indicate that selenite supplementation delays growth and maturation but not ultimate brood size. These brood size results are consistent with other *C. elegans* studies utilizing axenic media, which report brood sizes ranging from 20–51 [34], [35]. Thus the effect of Se on decreasing worm number is primarily due to impaired growth rather than a decrease in number of offspring.

### Selenate is less toxic than selenite or selenide in *C. elegans*

In higher animals, different oxidation states of Se exhibit different 50% lethal dose (LD_50_) concentrations [4], [5]. To determine if different inorganic forms would result in differential Se toxicity in *C. elegans*, wild-type N2 worms were cultured in axenic media supplemented with increasing concentrations of Se in the forms of sodium selenite, sodium selenate, or sodium selenide for 12 days (**Fig. 2**). With selenite, worm number is not affected until 0.1 mM Se. Concentrations greater than 1 mM selenite are lethal, with an LC_50_ value of 0.20±0.03 mM Se (**Fig. 2** **,** **Table 1**). Selenide results in Se toxicity that is virtually identical to selenite, with an LC_50_ of 0.23±0.05 mM Se. Supplementation with Se as selenate, however, significantly (*P*<0.001) shifts the Se response curve to the right (less toxic direction) with an LC_50_ of 0.95±0.02 mM Se. Supplementation with 0.1 or 0.25 mM Se as selenate is without effect, whereas 0.75 and 1.0 mM Se as selenate only reduce worm number to 65% and 51%, respectively, of the plateau levels; 2 mM Se as selenate is necessary to reduce worm number to <20% of plateau levels. Clearly, *C. elegans* metabolizes selenate to the toxic form(s) less readily than for selenite or selenide.

**Table 1:** LC_50_ values* for all strains with different inorganic Se forms.

	Selenite (mM Se)	Selenide (mM Se)	Selenate (mM Se)
N2	0.20±0.03^A^	0.23±0.05^A^	†0.95±0.02^B^
*trxr-1* (*−/−*)	0.21±0.02^A^	0.25±0.01^A^	†0.85±0.05^B^
*trxr-2* (*−/−*)	0.18±0.05^A^	0.21±0.01^A^	†0.76±0.05^B^
*dko*	0.16±0.02^A^	0.21±0.02^A^	†0.87±0.02^B^

* LC_50_ is the lethal concentration where total worm number is 50% of the maximal plateau worm number.

† Mean ± SEM;

A,B : means in a row with different letters are statistically different, *P*<0.05.

### ^75^Se incorporation into TRXR-1

**Figure 4:**
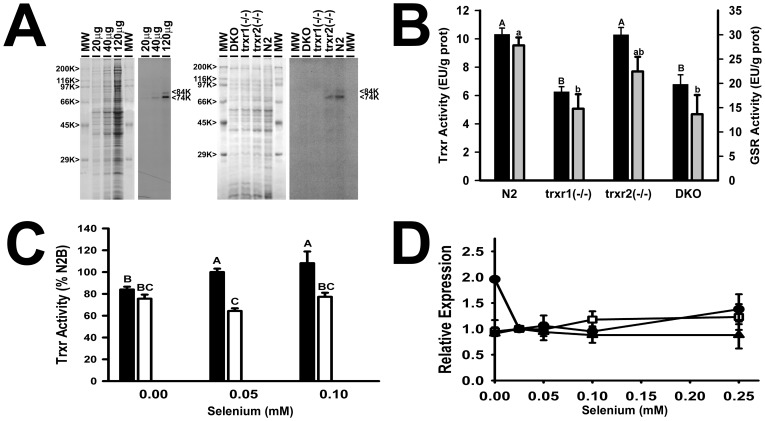
Effect of *trxr* deletions on ^75^Se selenoproteins, enzyme activity and mRNA levels. (A) Coomassie-blue stained gels and autoradiograms of SDS-PAGE of ^75^Se-labeled supernatants from *C. elegans* grown in axenic media containing 1 µCi [^75^Se]selenite/mL for 12 d. Left: N2 supernatant containing 20, 40, and 120 µg protein. Right: supernatant (50 µg protein) from N2, *trxr-1(−/−)*, *trxr-2(−/−)* and *dko*. Molecular weight (MW) standards were myosin, β-galactosidase, phosphorylase b, bovine albumin, chicken ovalbumin, and carbonic anhydrase. (B) Thioredoxin reductase (black bars) and glutathione reductase (gray bars) activity (EU/g protein) in N2 and *trxr* mutant strains cultured with 0.05 mM Se as Na_2_SeO_3_. (C) Se regulation of thioredoxin reductase activity in N2 (black bars) and *trxr-1* (*−/−*) (white bars) cultured in 0, 0.05 and 0.1 mM Se, expressed relative N2 cultured in 0.05 mM Se. (D) Se regulation of *trxr-1* (□), *trxr-2* (•) and *gsr-1* (▴) mRNA levels. N2 mRNA levels were assessed with 0–0.25 mM Se as selenite as normalized to eft-3 levels. Values are the mean ± SEM. A, B, C, a, b, c: Means with different letters are statistically different, *P*<0.05.

N2 *C. elegans* grown in axenic media for 12 days and supplemented with carrier-free [^75^Se]selenite readily incorporate ^75^Se into TRXR-1 (**Fig. 4A**). Autoradiography of 12-d supernatants shows a doublet with a major 74 kDa ^75^Se band, the predicted size of *C. elegans trxr-1* subunits (667 aa, 74,109 Da, NP_501085.2), along with a minor 84 kDa ^75^Se band. Similar doublets have been reported previously but not explained [20], [22]. These ^75^Se TRXR-1 subunits remain present in *trxr-2*(*−/−*) but both are missing as expected in *trxr-1*(*−/−*) and *dko* strains. This confirms that TRXR-1 is the sole selenoprotein in *C. elegans*. Importantly, these strains were grown axenically, in contrast to the previous reports, showing that the major and minor ^75^Se bands arise from the *trxr-1* gene and not from bacterial genes or bacterial Se metabolism.

### TRXR activity decreases in *trxr-1*(*−/−*) and *dko* strains

Attempts to assay *C. elegans* TRXR using the TXN-dependent activity assay [29] were unsuccessful using TXN from E. coli or Spirulina (T7915 or T3658, respectively, Sigma). TRXR activity was readily detected and measured, however, using human TXN (T8690, Sigma), suggesting that *C. elegans* TRXR is specific for eukaryotic TXN (data not shown). The human TRX-dependent TRXR specific activity (EU/g protein) of N2 grown for 12 days with 0.05 mM Se as selenite is 1/5^th^ of activity of Se adequate rat liver assayed under identical conditions. Loss of TRXR-1 (*trxr-1*(*−/−*), *dko*) results in a ∼40% decrease in TRXR activity as compared to N2. Surprisingly, this activity is not decreased in *trxr-2*(*−/−*) and is not further decreased in *dko* (**Fig. 4B**). Furthermore, TRXR activity in N2 grown axenically in the low Se basal media is 80% of the activity in N2 supplemented with 0.05 mM Se, and similar to TRXR activity levels in *trxr-1*(*−/−*) or *dko* strains (**Fig. 4C**). Supplementation with 0.1 mM Se does not increase TRXR activity above levels with 0.05 mM Se, indicating that Se incorporation into TRXR-1 plateaus at or before 0.05 mM Se. A partial explanation for only modest reduction in TRXR activity in Se-deficient media is that Trxr-2 mRNA transcript level is concomitantly increased 2-fold in N2 grown without Se supplementation (**Fig. 4D**). Knock-out of the *trxr-1* gene also results in an apparent 50% decrease in measured GSR activity in dialyzed, heat-denatured supernatants (**Fig. 4B**), suggesting that these reductases may have wider substrate specificity and/or readily contribute reducing equivalents capable of reducing oxidized GSSG.

### Deletion of *trxr-1*(−/−), *trxr-2*(−/−) or both does not modulate toxicity of selenite, selenide or selenate in *C. elegans*

**Figure 5:**
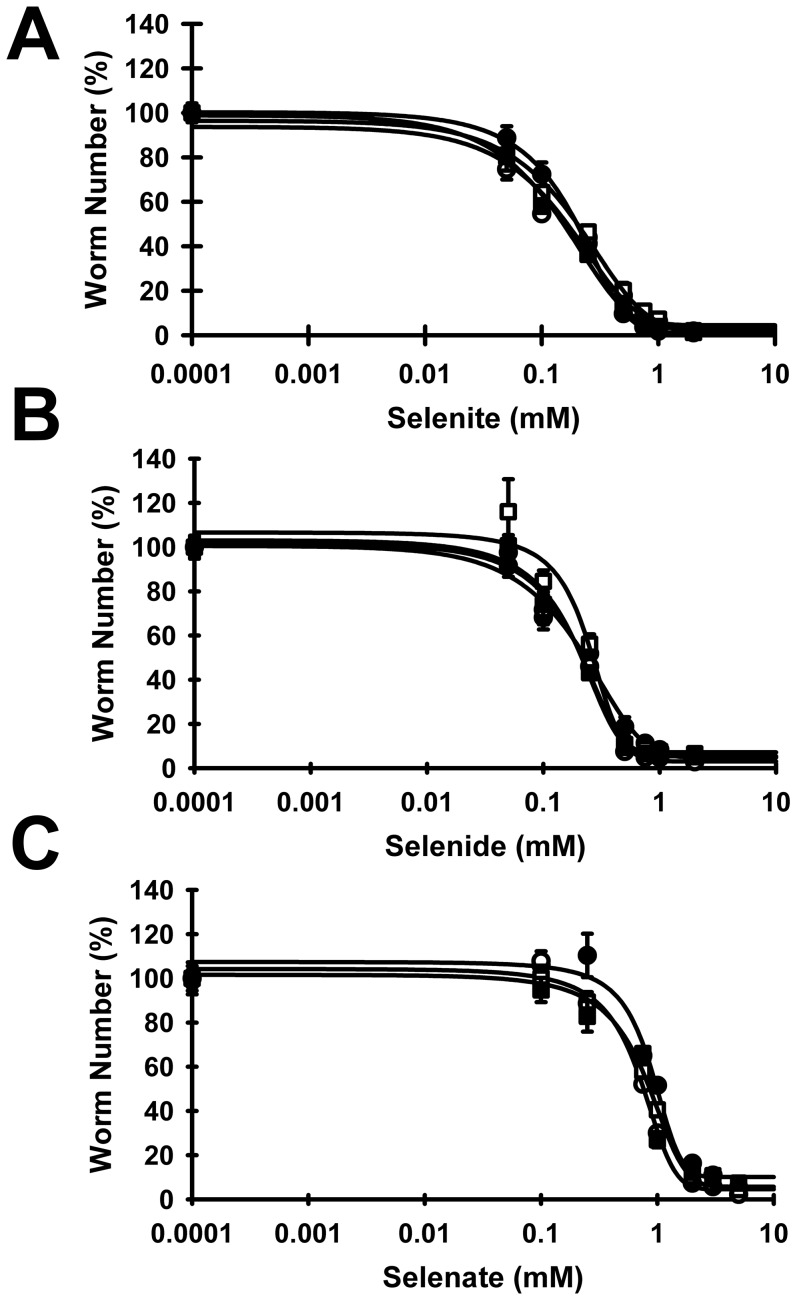
Thioredoxin reductase deletions do not modulate selenite, selenide, or selenate toxicity. N2 (•), *trxr-1(−/−)* (□), *trxr-2(−/−)* (○), and *dko* (▪) L1's were cultured with graded levels of selenite (A), selenide (B), and selenate (C) as described in Figure 4. Values represent the mean ± SEM, and lines are 4-parameter sigmoidal regressions for each Se form. For all response curves, no Se-supplemented mean was significantly higher than the 0 mM Se mean, P>0.05. The LC_50_ values for selenate were significantly different (*P*<0.001) from values for selenite and selenide, as shown in Table 1, indicating selenate is 1/4^th^ as toxic as selenite and selenide irrespective of *trxr* deletion.

To further study the role of TRXR in Se toxicity in *C. elegans*, N2, *trxr-1*(*−/−*), *trxr-*2(*−/−*), and *dko* strains were grown in axenic media with increasing selenite, selenate, or selenide concentrations (0–5 mM) for a period of 12 d. In ≥3 independent trials with each strain, with 3 replicates per trial, all strains were viable with no difference in susceptibility to selenium toxicity (**Figs. 5A, B, C**). Initial studies with the tm3462 *trxr-1*(*−/−*) strain were identical to those of the ok2580 *trxr-1*(*−/−*) strain, so only the ok2580 strain was used as the *trxr-1*(*−/−*) strain in further trials. As with N2, the *trxr-1*(*−/−*), *trxr-2*(*−/−*), and *dko* strains do not require Se for appropriate growth and reproduction. For selenite and selenide, the Se response curves of all deletion strains overlay the N2 Se response curves, with LC_50_ values for selenite and selenide for all null strains the same as N2, clearly indicating no significant change in protection against or susceptibility to Se toxicity. The selenate response curve observed in N2 was also recapitulated for the *trxr-1*(*−/−*), *trxr-2*(*−/−*), and *dko* strains, with LC_50_ values for selenate 4-fold higher than for selenite or selenide for all strains (**Table 1**). Clearly, loss of *trxr-1*, *trxr-2* or both does not modulate growth or Se toxicity for these inorganic forms of Se.

## Discussion

We used *C. elegans* grown axenically to develop a model to study metabolism of toxic levels of Se and the role of TRXR-1 in this process. The use of axenic media was important because bacteria synthesize Sec and incorporate it into selenoproteins, so bacterially-derived Se metabolites may otherwise provide necessary nutrients for *C. elegans* cultured with bacteria; for instance, culturing *C. elegans* with bacteria obscures the heme requirement of *C. elegans* [24]. In addition, the OP50 bacteria commonly used with *C. elegans* incorporate Se into selenoproteins [20]–[22], thus potentially concentrating Se from low-Se media and obscuring Se essentiality. The basal defined media also contained negligible Se, showing that *C. elegans* do not require Se under standard conditions. We found that neither Se nor TRXR-1 nor TRXR-2 is required for growth and reproduction of *C. elegans* in axenic media, and that loss of *trxr* expression does not modulate Se toxicity.

As predicted by gene sequences [36], Gladyshev [20] and Buettner [21] showed that *C. elegans* cultured with bacteria produce ^75^Se labeled TRXR-1 of the predicted size, plus a larger minor ^75^Se labeled band arising from *C. elegans* and not bacteria. Here using these bacteria-free cultures *and trxr-1* deletion strains, we confirm that TRXR-1 is the sole selenoprotein in *C. elegans*.

In pioneering studies by Stenvall and colleagues [22], deletion of *trxr-1* and *trxr-2* alone or in combination was without impact on *C. elegans*. Our studies reported here confirm those observations, and also show that Se deficiency is without effect in *C. elegans* grown axenically, indicating that loss of *trxr-1* genetically or nutritionally is without impact on growth and development. This group further showed that only with additional depletion of glutathione reductase (GSR-1) by RNAi did *trxr-1* deletion impact the *C. elegans* molting process; maintenance of cuticle oxidation state was disrupted by concurrent deletion of TRXR-1 and GSR-1, indicating that either one of these oxidoreductases alone is sufficient for proper disulfide reduction during molting. Similarly, Morgan and colleagues [3] found that glutaredoxin (*glrx-21*) deletion alone was without effect in *C. elegans* under standard conditions, but this deletion made *C. elegans* hypersensitive to the effect of selenite on movement and blocked the protective effect of exogenous glutathione. Interestingly, deletion of thioredoxin (TRX-1) itself reduces lifespan in *C. elegans* and increases sensitivity to oxidative stress [37], indicating TRX-1 is essential while TRXR-1 is not under standard conditions. Thus it appears in *C. elegans* that activities of TRXR, GSR-1 and GLRX-21 are complementary and overlapping, in contrast to mammals where single deletions of mammalian TXNRD1 and TXNRD2 [19] as well as TXN1 and TXN2 are embryonically lethal [38], [39].

The literature suggests that one role for TRXR is selenite reduction. Selenite is a substrate for mammalian TXNRD, reducing it to selenide with stoichiometric requirement for 3 NADPH [17]. Thus we hypothesized that deletion of *trxr-1* and/or *trxr-2* would modulate Se metabolism and toxicity in *C. elegans*. We found *trxr-1* deletion had no impact on selenite, as well as selenate toxicity, indicating that TRXR is not essential for metabolism of Se to toxic forms. In mammals, GSR provides an alternate selenite reduction pathway; while selenite is not a substrate for GSR, a combination of initial non-enzymatic reduction by GSH followed by reduction with GSR was observed in mammals, indicating that glutathione reductases can have a role in Se reduction [40]. *In vitro* studies using RNAi to silence human glutaredoxin-1 (GRX1) demonstrated that selenite, selenodiglutathione and selenocystine are all substrates for GRX1, and that GRX deletion reduces Se toxicity in this system [41]. In yeast, selenite sensitivity is increased with *grx1p* and *grx2p* mutant strains [42]. Interestingly, knockdown of TXNRD in mouse cancer cells enhances selenite toxicity, and this toxicity is accompanied by increased GSH secretion [43]. Collectively our study and these reports indicate that Se can be reduced by multiple enzyme systems, including TRXR, GRX and GSR, in *C. elegans* as well as in other organisms.

In rodent studies, dietary Se causes significant growth inhibition at levels of 3–5 µg Se/g diet [4], [44]. Se toxicity in our studies is due to a delay in growth that begins at day 6. To our knowledge this is the first report that Se toxicity decreases the rate of growth in *C. elegans*. In short-term acute studies with *C. elegans*, Morgan reported selenite was lethal to adults after 12 h with an LC_50_ of 3.47 mM Se [3], whereas we found a much lower LC_50_ of 0.20 mM Se as selenite in our model examining Se toxicity over the full lifespan including reproduction. Morgan further demonstrated selenite-induced lethality in their acute model was increased by H_2_O_2_ and reduced by exogenous GSH, but growth or development was not studied [3]. Stenvall showed that loss of TRXR-1 plus GSR-1 impaired molting at any of the four larval molts but did not report the effect of toxic Se on growth [22]. They also showed that deletion of *trxr-1* did not increase sensitivity to acute oxidative stress mediated by H_2_O_2_ or heat shock [22].

Our studies show that Se supplementation delays growth and maturation but not ultimate brood size. Time-course experiments indicate little difference in growth or reproduction of L1s supplemented with 0.2 mM selenite for the first 6 days, but delayed growth (length) and production of offspring due to supplemental Se were observed through day 12. Ultimately 0.2 mM selenite-supplemented worms have similar brood sizes to unsupplemented worms in these long-term, chronic Se supplementation studies. Higher concentrations of Se progressively impair growth and preclude reproduction; with 2 mM Se, L1 larva fail to develop beyond the L1 stage.

These are the first studies that examine the toxicity of different forms of Se in *C. elegans*. In lower forms of life, selenite, selenate and selenomethionine are all reported to be toxic [1]–[3], [33], [45]. In animals the toxicity of different inorganic Se forms is ranked: selenide (H_2_Se gas)>>selenite>selenate [4], [5], and organic forms such as selenomethionine and selenocysteine are less toxic than inorganic forms. Thus the four-fold decrease in *C. elegans* in selenate toxicity as compared to selenite and selenide toxicity is consistent with the toxicity observed in animal studies.

To be toxic Se needs to be absorbed. Selenate transport is typically rapid and appears to be mediated by sulfate transporters. Selenite uptake is largely mediated in bacteria by a sulfur permease [46], and in yeast by a monocarboxylate transporter (Jen1p) as gene deletion eliminates a major fraction of selenite uptake in these models [47]. In red blood cells (RBC), selenite is rapidly taken up by a distinctly different mechanism than selenate as selenate is not a competitive inhibitor of selenite transport [48], but the transporter has yet to be identified. We have observed that selenite uptake in *C. elegans* is a slow process, as carrier-free ^75^Se retention after 4 hours is <1% versus >30% after 30 sec in mouse RBCs (data not shown).

Selenate reduction to selenite occurs via common sulfur reduction pathways involving the ATP sulfurylase [45] in yeast, and this is thought to be the case at other phylogenetic levels [49]. Thus the reduced toxicity of selenate in our studies may be due to a slower rate of this first reduction. We found that deletion of both *trxr-1* and *trxr-2* in *C. elegans* had no effect on selenite toxicity as well as selenate and selenide toxicity. It should be noted that selenide in the presence of oxygen can cycle to more oxidized forms that are then re-reduced [10], [17]. Thus it is not surprising that selenite and selenide have equivalent toxicity response curves in our studies. Overall, no clear pattern emerges on the relative role of these oxidoreductases for protection against Se toxicity, as loss of *glrx* enhanced Se toxicity in *C. elegans* and yeast, but decreased Se toxicity in human cells, and loss of *trxr* increased Se toxicity in human cancer cells but had no effect in our studies in *C elegans*. A recent report indicates that S-adenosyl methionine can react with selenite or selenodiglutathione to spontaneously form methylselenol, and that methylselenol is a substrate for the thioredoxin and glutaredoxin systems, further documenting the overlapping roles of these systems on inorganic Se metabolism [50]. Collectively these experiments document an overlapping and intertwined set of enzymes that modulate Se toxicity by reducing selenite to selenide but also by influencing reduced thiol levels.

In summary, these studies show that *C. elegans* grown in axenic media have potential as a model for studying Se metabolism and toxicity. Absence of Se from the media or deletion of the lone *C. elegans* selenoprotein, TRXR-1, as well as TRXR-2, is without effect on growth and reproduction. Se is toxic, however, due to reduced growth rather than impaired reproduction, and loss of *trxr-1*, *trxr-2* or both does not modulate Se toxicity. Selenate is less toxic than selenite and selenide, and deletion of *trxr* is without effect for any of these forms, indicating that TRXR alone is not essential for Se reduction and production of Se toxicity in this model. Future use of additional deletions in *C. elegans* offers the potential to better unravel the metabolism underlying Se toxicity.
